# Evaluation of Treatment Modalities and Oncologic Outcomes in Hand Soft Tissue Sarcomas—A Systematic Review of the Literature

**DOI:** 10.3390/cancers17193204

**Published:** 2025-10-01

**Authors:** W. Rodrigo Calmet Rocca, Rayna S. Kuthiala, Marcos R. Gonzalez, Juan Pretell-Mazzini

**Affiliations:** 1 Facultad de Medicina Alberto Hurtado, Universidad Peruana Cayetano Heredia, Lima 15102, Peru; 2 Musculoskeletal Oncology Service, Department of Orthopaedic Surgery, Massachusetts General Hospital, Harvard Medical School, Boston, MA 02115, USAmgonzalez52@mgh.harvard.edu (M.R.G.); 3 Tufts Medical Center Orthopaedic Residency Program, Tufts University School of Medicine, Boston, MA 02111, USA; 4 Miami Cancer Institute, Division of Orthopedic Oncology, Baptist Health System South Florida, Plantation, FL 33324, USA

**Keywords:** hand sarcoma, soft tissue sarcoma, metastasis, local recurrence, overall survival, systematic review

## Abstract

**Simple Summary:**

Cancers that develop in the soft tissues of the hand are very rare, but they can easily be mistaken for harmless conditions. Because of their small size and unusual appearance, many patients first undergo surgery without knowing the true diagnosis, which can delay proper treatment affecting long-term outcomes. We reviewed 18 studies that collectively included more than 500 patients with soft tissue sarcomas of the hand to better understand how these tumors present, how they are treated, and what results patients can expect. We found that many patients needed additional surgery, and in some cases, amputation was also required. Radiation and chemotherapy were also used for treatment, and while overall survival was encouraging, the risk of cancer returning or spreading remained significant. By summarizing what is currently known, this research highlights the need for early recognition and specialized care to improve results for people with soft tissue cancers of the hand.

**Abstract:**

Background/Objectives: Soft tissue sarcomas (STS) of the hand are rare, representing only 2% of all STS. The small size and benign appearance of these tumors often lead to unplanned excisions and diagnostic delay. This systematic review sought to characterize the clinical presentation, histology, treatment modalities, and oncological outcomes of hand STS. Methods: A systematic review of PubMed and Embase was conducted following PRISMA guidelines. The protocol was registered on PROSPERO. We included studies with ≥10 patients with STS that provided data on treatment options and oncologic outcomes. Data was extracted regarding demographics, tumor features, treatment modalities, and survival metrics. Results: Eighteen studies comprising 570 patients were included. Most tumors were <5 cm, and 56.8% were deep (subfascial). Epithelioid and synovial sarcomas were the most common histologies, accounting for 27% and 17% of cases, respectively. UEs were seen in 57% of cases, and 26% of patients required amputation. Positive surgical margins were reported in 16% of patients. Radiation therapy and chemotherapy were used in 40% and 17% of patients, respectively. Twelve and 15% of patients developed regional lymph node and distant metastases, respectively. Local recurrence occurred in 20% of cases. Five- and ten-year overall survival were 80% and 77%, respectively. Disease-free survival at those time points were 77% and 74%, respectively. Conclusions: Hand STSs are challenging due to their rarity, small size, and high rates of UEs. Despite favorable survival rates, local recurrence and metastases remain a concern. Early referral to specialized centers and individualized treatment strategies are essential for improving outcomes.

## 1. Introduction

Soft tissue sarcomas (STS) are rare tumors, accounting for less than 1% of all malignant neoplasms in the United States, with approximately 13,000 new cases reported annually [1,2,3]. Of these, only 13% arise in the upper extremity, and just 2% involve the hand [4]. Over the past three decades, the prognosis for limb sarcomas has significantly improved, driven by advances in imaging, surgical techniques, and adjuvant therapies [5]. Notably, wide resection has been shown to achieve survival outcomes comparable to amputations while preserving better limb function [5,6]. Despite these advances, the rarity of STS—and particularly of hand STS—has limited the development of high-quality guidelines for optimal management. As a result, up to 53% of STS might be excised without consideration for oncological principles, under the assumption that these masses are benign [5,6,7]. This issue is particularly pronounced in the hand, where sarcomas are infrequently considered in the differential diagnosis, leading to high rates of unplanned excision (UE).

The management of STS is particularly complex in the hand due to its intricate anatomy, compact compartments, and high density of critical structures. Therefore, achieving oncologic control without compromising function is highly challenging, and re-excision following UE carries significant morbidity. Further complicating treatment decisions is the poorly defined role of radiation therapy (RT) and chemotherapy in this population. Although RT is a cornerstone of STS management at other anatomic sites, its use in the hand is associated with fibrosis and functional impairment—even when the limb is preserved [1,8]. Similarly, although chemotherapy has shown survival benefits in metastatic disease, its efficacy in localized hand STS remains uncertain [9].

Beyond these clinical challenges, real-world barriers such as limited access to specialized sarcoma centers, the high cost of advanced imaging, and lack of awareness among primary physicians further contribute to delays in diagnosis and suboptimal initial management [5].

Given these challenges, our study sought to evaluate therapeutic strategies employed in patients with hand STS and their associated oncologic outcomes.

## 2. Materials and Methods

### 2.1. Search Strategy

We conducted a systematic review following the Preferred Reporting Items for Systematic Reviews and Meta-Analyses (PRISMA) guidelines. A comprehensive search of the PubMed and Embase databases from inception to June 30th, 2025 was performed. The following search terms were used: (“soft tissue sarcoma*” OR “soft-tissue sarcoma” OR “epithelioid sarcoma*” OR “synovial sarcoma*” OR “hand sarcoma*” OR “sarcoma* of the hand*”) AND (hand). Additionally, the reference lists of included studies were manually screened to identify any relevant articles missed in the initial search. The protocol for this systematic review was registered on PROSPERO CRD420250618076.

### 2.2. Eligibility Criteria

To be included in our study, articles had to meet the following criteria based on the PICOS framework: the Population (P) consisted of patients with histologically confirmed soft tissue sarcomas of the hand; the Interventions (I) included surgical treatment (limb salvage or amputation) and/or adjuvant or neoadjuvant therapies (radiation therapy and/or systemic therapy); the Comparisons (C)**,** when available, involved different therapeutic strategies (e.g., limb salvage vs. amputation, with or without adjuvant treatment); and the Outcomes (O) included oncologic endpoints such as local recurrence, metastasis, overall survival, and disease-free survival. Eligible Study designs (S) were retrospective or prospective cohort studies with a minimum of 10 patients. We excluded case reports, non-peer-reviewed publications (e.g., conference proceedings, preprints), non-human studies, and articles not written in English, Spanish, Italian, French, or German. No additional filters were applied.

### 2.3. Selection, Data Collection, and Extraction Process

The search was separately conducted in both databases, and the results were exported to Covidence™ (Veritas Health Information, Deerfield, IL, USA). After removing duplicates, two reviewers (R.K. and R.C.) independently screened the studies for eligibility. In case of disagreement, the senior author (J.P.) was consulted, and the final decision was reached by consensus.

Data was extracted using a standardized sheet. The following demographic and clinical variables were collected: study sample, patient age, tumor location within the hand, tumor size and depth, histology subtype, histologic grade, extent of disease at presentation (localized versus metastatic), and follow-up duration. With regard to treatment modalities, we extracted the following variables: prior UE, use of (neo)-adjuvant radiation therapy (RT) or chemotherapy (QT), type of surgery (limb salvage versus amputation), and final margin status. The following oncologic outcomes were obtained: regional and distant metastasis and local recurrence rates, and overall and disease-free survival at 2, 5, and 10 years from initial surgery. Regional metastasis referred to involvement of lymph nodes draining the area where the tumor is located.

### 2.4. Study Selection and Patient Characteristics

**Figure 1:**
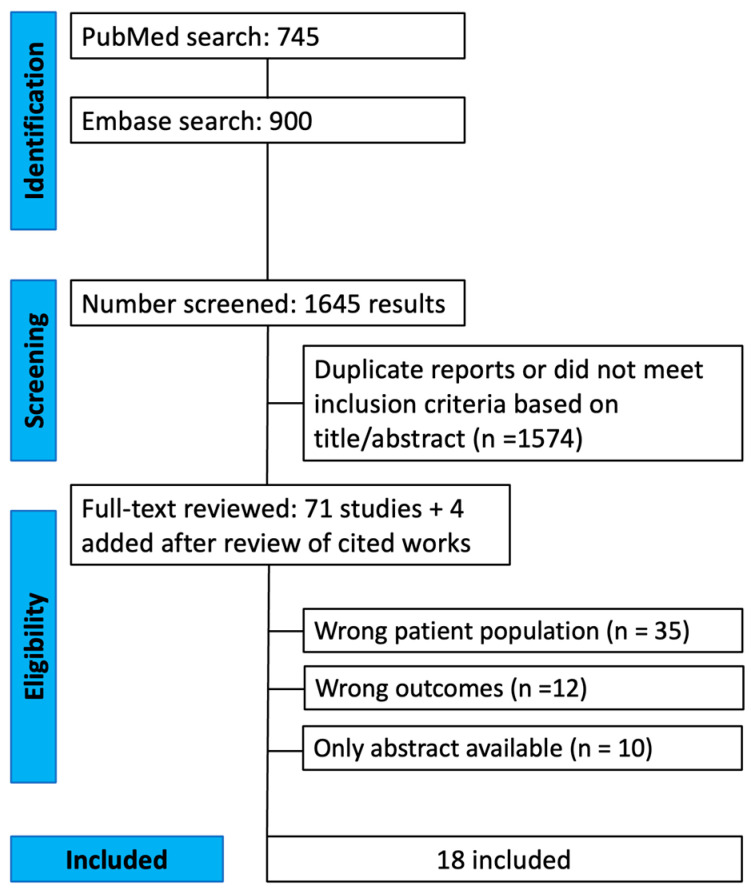
PRISMA Flowchart for patient inclusion.

Our search query yielded 745 articles from PubMed and 900 from Embase (Figure 1). After removing 100 duplicates, the titles and abstracts of 1545 unique studies were screened, and 1368 studies were excluded. The full texts of the remaining 70 manuscripts were then reviewed, 4 were added after review of cited works. 56 studies were excluded due to wrong patient population, wrong outcomes, patients overlapping between studies or manuscripts not found. Eighteen articles were finally included in our review [1,5,8,10,11,12,13,14,15,16,17,18,19,20,21,22,23,24]. The 18 included studies comprised 570 patients, with the study sample ranging from 10 [8,22] to 109 [1]. The mean age at diagnosis ranged from 18 [10] to 55 years [11], with most studies reporting mean ages between the third and fifth decade of life (Table 1). A male predominance was observed in most cohorts, though six studies reported more female patients [1,14,16,20,22,24]. Tumor size at diagnosis varied from 2.2 cm [12] to 5 cm [13], and the proportion of high-grade tumors ranged from 47% [16] to 94% [5]. At diagnosis, the proportion of localized (non-metastatic) tumors ranged from 83% [13] to 100% [5,11,14]. The mean follow-up duration reported by studies ranged from 2 years [16] to 14.7 years [24].

Epithelioid sarcoma was the most frequently reported histologic subtype, accounting for 28% of cases, followed by synovial sarcoma (17%), fibrosarcoma (5%), and undifferentiated pleomorphic sarcoma (5%) (Table 2).

### 2.5. Assessment of Study Quality

Quality assessment was performed using a modified version of the Strengthening the Reporting of Observational Studies in Epidemiology (STROBE) checklist [25]. We selected 10 of the 22 STROBE items most relevant to our research question, following an approach similar to previous systematic reviews in the orthopedic oncology literature [7,9]. Each item was scored as “well reported” (2 points), “partially reported” (1 point), or “poorly reported/absent” (0 points), with a maximum possible score of 20. Based on total scores, studies were classified as high quality (≥14), moderate quality (7–13), or low quality (≤6). These quality assessments were used to contextualize our findings and identify methodological limitations across the included studies. A detailed breakdown of the scores for each article is provided in Table 3.

### 2.6. Statistical Analysis

Demographic, clinical, and tumor characteristics are presented in narrative form. Continuous variables were displayed as means or medians depending on the metric reported by the authors. For categorical variables, we quantified the proportion of patients that presented a certain event over the eligible patient population. We calculated weighted proportions to adjust the values in question to the sample size of each study. All analyses were performed using Stata Special Edition 14 (StataCorp, College Station, TX, USA).

Although our review followed systematic review methodology, a quantitative meta-analysis was not conducted. Most of the included studies were retrospective cohorts or case series that did not compare interventions or include control groups. As such, data were primarily descriptive, and pooling results for a meta-analysis was not methodologically appropriate.

## 3. Results

### 3.1. Surgical Management

UE was present in 57% of patients, with studies reporting rates ranging from 16% [14] to 100% [10] (Table 4). Amputation was performed in 26% of patients, with studies reporting amputation rates ranging from 0% [11,12,22] to 88% [23]. Positive surgical margins were reported in 16% of cases, with variation from 0% [20] to 42% [14].

### 3.2. (Neo)-Adjuvant Therapy

RT was administered to 40% of patients, ranging from 8% [11] (11) to 61% [10]. Chemotherapy was administered to 17% of patients, ranging from 0% [22] to 80% [8] (Table 4).

### 3.3. Oncologic Outcomes

Patients developed regional lymph node metastasis in 12% of cases, with reported rates by studies ranging from 0% [8,11,22] to 63% [11,22] (Table 5) [24]. Distant metastasis occurred in 15% of cases, with studies reporting rates ranging from 0% [24] to 60% [8] Local recurrence was observed in 20% of patients, with reported rates between 4% [1,23] and 61% [10].

Overall survival was 92% at 2 years, 80% at 5 years, and 77% at 10 years. Disease-free survival was 79% at 2 years, 77% at 5 years, and 74% at 10 years.

## 4. Discussion

STS of the hand are exceptionally rare, comprising approximately 2% of all STS cases, with an estimated incidence of just one per 1,250,000 individuals each year [14]. Often presenting as small, painless masses, they frequently lack distinctive clinical features, making early diagnosis particularly challenging. To the best of our knowledge, this is the first systematic review to assess the treatment modalities and oncologic outcomes of patients with STS of the hand.

A major challenge in managing STS of the hand is the high rate of UEs, observed in 57% of patients in our cohort. Although referral to specialized sarcoma centers is recommended, benign soft tissue tumors account for 99% hand lesions [5,26,27], leading to frequent misdiagnosis and delays in advanced imaging or biopsy. Common benign lesions—such as synovial cysts, mucoid cysts, and tenosynovial giant cell tumors—often present with characteristic clinical and radiologic features, which can further reduce clinical suspicion for malignant lesions [5]. Nevertheless, the prevalence of UEs in our cohort remained high, with three studies reporting rates of 72%, 81%, and 100% [10,17,18]. The predominance of cases diagnosed in patients under age 55 further highlights the need for clinical vigilance, even in younger populations. While guidelines recommend early referral for lesions > 5 cm (or >3 cm in the hand), deep or rapidly growing [4,27,28,29], 72% of tumors in our series measured <5 cm, contributing to delayed recognition and suboptimal initial management.

Margin status remains a critical prognostic factor in soft tissue sarcomas (STS). In our cohort, 16% of patients had positive surgical margins following resection, which was associated with a significantly increased risk of adverse outcomes, including a 12-fold increase in local recurrence, a 3-fold higher risk of metastasis, and a 5-fold increase in mortality [14]. Interestingly, despite the relatively high rate of positive margins, the overall incidence of metastasis in our cohort was only 15%, which is comparable to rates reported for sarcomas in other anatomical sites, such as the extremities [30]. This discrepancy between margin status and survival outcomes warrants further discussion, as it may reflect the influence of additional factors such as tumor biology or early detection.

Several studies have underscored the importance of achieving negative margins, particularly in extremity sarcomas [13,14,20], emphasizing the need for careful preoperative planning. Historically, limb-sparing surgery was underutilized in hand STS due to concerns about local recurrence and functional impairment [15,18]. However, our data indicate that limb-sparing procedures were utilized in 76% of cases, highlighting a trend toward prioritizing functional preservation while maintaining effective oncologic control.

RT and chemotherapy were administered in 40% and 17% of patients in our cohort, respectively. While RT is a cornerstone of treatment for extremity STS, its use in the hand is complicated by a higher risk of complications, including fibrosis, wound healing issues, and functional deficits [31]. Notably, perioperative RT in the hand has been associated with complication rates of up to 73% [32]. This rate is significantly higher than the rates previously reported in other upper and lower extremity sites, which ranges from 26% to 44% [33,34,35].

The significantly higher complication rate at this anatomic site underscores the importance of a distinct risk-benefit assessment. Clinical experience with proton therapy for extremity sarcomas, including the hand, is limited but suggests comparable local control to photon therapy, with potential advantages in reducing late toxicity and preserving function, particularly in younger patients since it reduces radiation exposure to healthy tissue [36,37]. Nevertheless, when carefully selected, RT remains a valuable adjunct to improve local control while striving to preserve function; however, it may not be required when negative-margin resections are achieved, such as with ray or transradial amputation. The role of chemotherapy in hand STS is less well defined. While its benefit in improving overall survival in metastatic disease is well established [9,31,38], its utility in localized disease—relevant to most patients in our cohort—remains controversial. Even though multiple meta-analyses have shown a benefit of doxorubicin-based chemotherapy in improving overall survival in localized, resectable STS, findings across the included studies have been inconsistent, with most of them not demonstrating a significant survival advantage [9,39,40]. Therefore, physicians should evaluate on a case-by-case basis the need to use RT and chemotherapy and include factors such as the expected activity level of patients, tumor histology, and the likelihood of achieving margin-negative resections into the decision-making process.

Oncologic outcomes for STS of the hand showed substantial variability across studies. In first place, at the moment of diagnosis, 47% [5,16] to 94% [5] of the tumors were considered high grade. Additionally, regional and distant metastases occurred in 12% and 15% of patients, respectively. The occurrence of the former was more frequent in epithelioid and synovial sarcomas, in agreement with previous studies [17]. However, it is important to emphasize that tumors of the lower extremity generally exhibit higher rates of distant metastasis compared to those in the upper extremity, likely due to their larger size and deeper location at the time of diagnosis [41,42].

Finally, local recurrence was observed in 20% (range: 4 to 61%) of patients. Tumor size at presentation was predominantly less than 5 cm, consistent with prior reports. Although a diameter of 5 cm is widely cited as a threshold for increased metastatic risk and mortality [20,30,43,44,45,46,47,48] many hand STS fall below this size cutoff yet still exhibit aggressive biological behavior and remain associated with poorer survival and higher local recurrence rates [19,49]. Tumor depth, another important prognostic factor, has been correlated with increased risks of metastasis, recurrence, and disease-specific mortality [20] notably, over half of tumors in our cohort (56.84%) were located in deep (subfascial) planes. These findings underscore a critical limitation of the current American Joint Committee on Cancer (AJCC) staging system, which heavily emphasizes tumor size but does not account for depth. In hand STS, where tumors are typically small, AJCC staging alone may be insufficient to accurately stratify risk. Additional parameters such as histological subtype, grade and depth should be considered essential components of risk assessment and treatment planning. Overall survival in our cohort was 92% at 2 years, 80% at 5 years, and 77% at 10 years; disease-free survival was 79%, 77%, and 74%, respectively. Despite advances in treatment, the risks of recurrence, metastasis, and mortality remain considerable, highlighting the need for a more nuanced approach to prognostication in hand STS.

This study has several limitations. First, the rarity of hand STS and the reliance on small, retrospective case series introduce selection bias and limit the generalizability of our findings. Secondly, included studies had different treatment protocols, and follow-up durations, making direct comparisons challenging. Third, important oncologic variables such as tumor grade, depth, margin status, type of amputation, metastasis and survival information were not consistently reported across all studies, which may have impacted our pooled analysis. Finally, some studies did not differentiate between primary resections and unplanned excisions, which could have confounded the oncologic outcomes reported and introduced further heterogeneity in the analysis.

High rates of unplanned excision (57%) and the typically small size of hand soft tissue sarcomas (STS) complicate timely diagnosis and optimal initial management. Despite being a standard component of treatment for appendicular STS, neoadjuvant or adjuvant radiotherapy was utilized in less than half of cases, reflecting concerns about potential functional impairment in the hand. While long-term survival is relatively favorable, both local recurrence and distant metastasis remain substantial risks, affecting up to 20% of patients.

## 5. Conclusions

Soft tissue sarcomas of the hand are rare malignancies that often mimic benign lesions, contributing to frequent unplanned excisions. In our review, local recurrence occurred in about 20% of patients, distant metastasis in 15%, and overall survival was approximately 80% at 5 years. Achieving negative margins is recommended to improve oncologic outcomes, but surgical strategies must also prioritize functional preservation. Early referral to specialized sarcoma centers and use of risk stratification models incorporating histology, depth, and size may support more tailored management. Given the rarity of hand STS and reliance on retrospective series, multicenter collaborations and standardized reporting are needed to strengthen the evidence base.

Emerging tools such as diffusion-weighted MRI and PET/CT may enhance early detection and surgical planning in hand STS [50]. Advances in microsurgical reconstruction also offer opportunities to achieve negative margins while preserving function [51]. Collaborative multicenter studies integrating these innovations are needed to establish standardized management strategies.

## Figures and Tables

**Table 1:** Baseline characteristics of patients.

Author	Sample (*n*)	Mean Age (years)	M:F Ratio	Location	Mean Tumor Size (cm)	Deep (Sub-Fascial)	Localized	Metastatic	High-Grade	Mean FU (Years)
Chapman et al. [1]	109	36	0.98	-	2.1 ^α^	88%	96.3%	3.7%	64%	6.1
Farzaliyev et al. [17]	23	27	1.3	W (48%), MC (35%), P (17%)	-	-	-	-	-	7
Thumser et al. [5]	16	40	1.67	-	5	-	100.0%	0.0%	94%	6
Dadras et al. [12]	51	41	1.21	MC (47%), W (35%), P (18%)	2.2	64%	-	-	69%	6.5 ^α^
Lans et al. [18]	64	46	-	MC (64%), P (28%), C (8%)	3	27%	-	-	70%	4
Dean et al. [19]	26	50	1.36	-	2.7 α	-	-	-	67%	4.9
Nicholson et al. [16]	17	45	0.88	C (52%), W (30%), P (18%)	2.2	-	94.0%	6.0%	47%	2
Houdek et al. [20]	46	38	0.91	C or W (56%), P (44%)	-	40%	91.4%	8.6%	-	5
Mirous et al. [21]	16	49	3	W (56%), MC (44%)	-	-	-	-	63%	4.5
Wang et al. [11]	13	55	1.1	-	-	-	100.0%	0.0%	-	4
Deroose et al. [22]	10	51	0.66	C (60%), W (30%), P (10%)	-	-	-	-	80%	3
Pradhan et al. [14]	63	45	0.43	MC (43%), P (25%), C (22%)	4	45%	100.0%	0.0%	68%	4
Michal et al. [24]	13	32	0.625	C (38%), MC (32%), P (30%)	1	-	-	-	-	14.7
Herr et al. [23]	28	31	1.8	P (53%), C (25%), W (21%)	2.5 ^α^	-	-	-	-	10
McPhee et al. [15]	24	39	2	W (50%), C (33%), P (16%)	-	-	80.0%	20.0%	91%	7
Gross et al. [10]	18	18	1.57	MC (88%), P (12%)	-	-	-	-	78%	5.5
Brien et al. [13]	23	31	1.3	P (47%), C (34%), W (17%), MC (2%)	-	65%	83.0%	17.0%	86%	3
Prat et al. [8]	10	28	4	MC (60%), P (40%)	2.6	5%	-	-	-	8

Symbols: ^α^ Refers to median value. Abbreviations: C: carpal; F: female; FU: follow-up; M:F: male-to-female; MC: metacarpal; P: phalangeal; W: wrist.

**Table 2:** Most common STS subtypes in the hand and wrist.

Author (Year)	Sample (*n*)	ES	SS	UPS	FS	LMS	MFS	Other
Chapman et al. [1]	109	25%	16%	4%	4%	5%	7%	40%
Farzaliyev et al. [17]	23	100%	0%	0%	0%	0%	0%	0%
Thumser et al. [5]	16	31%	19%	0%	0%	6%	6%	38%
Dadras et al. [12]	51	14%	26%	10%	2%	6%	6%	35%
Lans et al. [18]	64	20%	16%	13%	11%	5%	0%	38%
Dean et al. [19]	26	35%	8%	8%	0%	12%	15%	23%
Nicholson et al. [16]	17	6%	18%	0%	0%	12%	18%	47%
Houdek et al. [20]	46	22%	17%	4%	15%	7%	0%	35%
Mirous et al. [21]	16	13%	19%	6%	0%	13%	6%	44%
Wang et al. [11]	13	8%	15%	0%	23%	15%	0%	39%
Deroose et al. [22]	10	50%	20%	0%	0%	0%	0%	30%
Pradhan et al. [14]	63	18%	16%	0%	11%	5%	0%	51%
Michal et al. [24]	13	0%	100%	0%	0%	0%	0%	0%
Herr et al. [23]	28	100%	0%	0%	0%	0%	0%	0%
McPhee et al. [15]	24	25%	8%	0%	0%	0%	0%	67%
Gross et al. [10]	18	11%	6%	0%	0%	0%	0%	83%
Brien et al. [13]	23	9%	35%	13%	0%	13%	0%	30%
Prat et al. [8]	10	100%	0%	0%	0%	0%	0%	0%
**Weighted percentage**	**570**	**28%**	**17%**	**4%**	**5%**	**5%**	**4%**	**37%**

ES: epithelioid sarcoma; FS: fibrosarcoma; LMS: leiomyosarcoma; MFS: myxofibrosarcoma; SS: synovial sarcoma; UPS: undifferentiated pleomorphic sarcoma.

**Table 3:** Quality assessment using the STROBE checklist.

Author	Setting	Participants	Variables	Data Sources	Statistical Methods	Participants	Descriptive Data	Outcome Data	Main Results	Limitations	Score
Chapman et al. [1]	2	2	2	1	2	2	2	2	2	2	19
Farzaliyev et al. [17]	2	2	2	1	2	2	2	2	2	1	18
Thumser et al. [5]	2	2	2	1	1	2	1	2	2	2	17
Dadras et al. [12]	2	2	1	1	1	2	2	2	2	1	16
Lans et al. [18]	2	2	1	2	2	2	2	2	2	1	18
Dean et al. [19]	2	2	2	1	1	2	2	2	1	1	16
Nicholson et al. [16]	2	2	1	2	1	2	2	2	2	0	16
Houdek et al. [20]	2	2	1	2	1	2	2	2	1	2	17
Mirous et al. [21]	2	2	1	1	1	2	2	2	2	1	16
Wang et al. [11]	2	2	2	2	1	2	2	2	2	1	18
Deroose et al. [22]	2	2	2	1	2	2	2	2	2	1	18
Pradhan et al. [14]	2	2	1	1	1	2	2	2	2	1	16
Michal et al. [24]	2	2	2	2	1	2	2	1	1	1	16
Herr et al. [23]	2	2	1	2	1	2	2	2	2	1	17
McPhee et al. [15]	2	2	2	1	1	2	2	2	2	1	17
Gross et al. [10]	2	2	1	1	1	2	2	2	2	1	16
Brien et al. [13]	2	2	1	2	1	2	2	2	2	1	17
Prat et al. [8]	2	2	2	1	1	2	2	2	2	1	17

STROBE: Strengthening the Reporting of Observational Studies in Epidemiology.

**Table 4:** Management of hand and wrist STS.

Author	Prior UE	RT	QT	LSS	Amputation	Positive Margins
Chapman et al. [1]	-	33%	12%	54%	46%	6%
Farzaliyev et al. [17]	87%	52%	22%	87%	13%	30%
Thumser et al. [5]	-	50%	70%	30%	70%	6%
Dadras et al. [12]	67%	33%	25%	100%	0%	12%
Lans et al. [18]	81%	59%	6%	98%	2%	14%
Dean et al. [19]	46%	27%	4%	73%	27%	-
Nicholson et al. [16]	41%	59%	8%	100%	0%	-
Houdek et al. [20]	72%	52%	24%	87%	13%	0%
Mirous et al. [21]	63%	56%	31%	100%	0%	-
Wang et al. [11]	39%	8%	38%	100%	0%	-
Deroose et al. [22]	40%	40%	0%	100%	0%	30%
Pradhan et al. [14]	16%	36%	4%	71%	29%	42%
Michal et al. [24]	-	38%	8%	-	8%	-
Herr et al. [23]	35%	21%	7%	15%	88%	12%
McPhee et al. [15]	-	16%	13%	60%	40%	-
Gross et al. [10]	100%	61%	11%	72%	28%	22%
Brien et al. [13]	-	60%	34%	66%	34%	34%
Prat et al. [8]	-	30%	80%	90%	10%	10%
**Weighted percentage**	**57%**	**40%**	**17%**	**74%**	**26%**	**16%**

LSS: limb salvage surgery; QT: chemotherapy; RT: radiation therapy; STS: soft tissue sarcoma; UE: unplanned excision.

**Table 5:** Oncologic outcomes of patients with hand and wrist STS.

Author	Metastasis	Local Recurrence	Overall Survival			Disease-Free Survival		
			2-Year	5-Year	10-Year	2-Year	5-Year	10-Year
Chapman et al. [1]	6%	4%	100%	95%	92%	95%	89%	88%
Farzaliyev et al. [17]	39%	30%	-	-	-	-	90%	62%
Thumser et al. [5]	43%	12%	87%	56%	13%	-	-	-
Dadras et al. [12]	16%	35%	-	-	91%	-	91%	91%
Lans et al. [18]	23%	13%	-	83%	83%	-	69%	65%
Dean et al. [19]	27%	19%	-	86%	-	-	-	-
Nicholson et al. [16]	29%	12%	92%	-	-	62%	-	-
Houdek et al. [20]	30%	11%	96%	78%	72%	71%	63%	63%
Mirous et al. [21]	6%	13%	-	94%	-	-	-	-
Wang et al. [11]	8%	8%	92%	-	-	-	85%	-
Deroose et al. [22]	-	30%	90%	60%	60%	-	-	-
Pradhan et al. [14]	22%	32%	-	87%	71%	-	-	-
Michal et al. [24]	0%	15%	-	-	-	-	-	-
Herr et al. [23]	32%	4%	-	85%	85%	-	67%	67%
McPhee et al. [15]	25%	41%	-	59%	53%	-	-	-
Gross et al. [10]	39%	61%	61%	55%	55%	50%	50%	50%
Brien et al. [13]	17%	35%	93%	50%	-	-	-	-
Prat et al. [8]	50%	50%	60%	50%	40%	30%	30%	30%
**Weighted percentage**	**21%**	**20%**	**92%**	**80%**	**77%**	**79%**	**77%**	**74%**

STS: soft tissue sarcoma.

## Data Availability

The original contributions presented in this study are included in the article. Further inquiries can be directed to the corresponding author.

## Abbreviations

The following abbreviations are used in this manuscript:

C	Carpal
F	Female
FU	Follow-up
M:F	Male-to-Female
MC	Metacarpal
P	Phalangeal
W	Wrist
ES	Epithelioid Sarcoma
FS	Fibrosarcoma
LMS	Leiomyosarcoma
MFS	Myxofibrosarcoma
SS	Synovial Sarcoma
UPS	Undifferentiated Pleomorphic Sarcoma
STROBE	Strengthening the Reporting of Observational Studies in Epidemiology
LSS	Limb Salvage Surgery
QT	Chemotherapy
RT	Radiotherapy
STS	Soft Tissue Sarcoma
UE	Unplanned Excision
